# The effects of training population design on genomic prediction accuracy in wheat

**DOI:** 10.1007/s00122-019-03327-y

**Published:** 2019-03-19

**Authors:** Stefan McKinnon Edwards, Jaap B. Buntjer, Robert Jackson, Alison R. Bentley, Jacob Lage, Ed Byrne, Chris Burt, Peter Jack, Simon Berry, Edward Flatman, Bruno Poupard, Stephen Smith, Charlotte Hayes, R. Chris Gaynor, Gregor Gorjanc, Phil Howell, Eric Ober, Ian J. Mackay, John M. Hickey

**Affiliations:** 1 The Roslin Institute and Royal (Dick) School of Veterinary Studies, The University of Edinburgh, Easter Bush, Midlothian, Scotland UK; 2 The John Bingham Laboratory, NIAB, Huntingdon Road, Cambridge, CB3 0LE UK; 3 KWS UK Ltd, 56 Church Street, Hertfordshire, SG8 7RE UK; 4 RAGT UK, Grange Rd, Saffron Walden, CB10 1TA UK; 5 Limagrain UK Ltd, Rothwell, Market Rasen, Lincolnshire, LN7 6DT UK; 6 Elsoms Wheat Limited, Pinchbeck Road, Spalding, Linconshire, PE11 1QG UK; 7 IMplant Consultancy Ltd., Chelmsford, UK

## Abstract

Genomic selection offers several routes for increasing the genetic gain or efficiency of plant breeding programmes. In various species of livestock, there is empirical evidence of increased rates of genetic gain from the use of genomic selection to target different aspects of the breeder’s equation. Accurate predictions of genomic breeding value are central to this, and the design of training sets is in turn central to achieving sufficient levels of accuracy. In summary, small numbers of close relatives and very large numbers of distant relatives are expected to enable predictions with higher accuracy. To quantify the effect of some of the properties of training sets on the accuracy of genomic selection in crops, we performed an extensive field-based winter wheat trial. In summary, this trial involved the construction of 44 F_2:4_ bi- and tri-parental populations, from which 2992 lines were grown on four field locations and yield was measured. For each line, genotype data were generated for 25 K segregating SNP markers. The overall heritability of yield was estimated to 0.65, and estimates within individual families ranged between 0.10 and 0.85. Genomic prediction accuracies of yield BLUEs were 0.125–0.127 using two different cross-validation approaches and generally increased with training set size. Using related crosses in training and validation sets generally resulted in higher prediction accuracies than using unrelated crosses. The results of this study emphasise the importance of the training panel design in relation to the genetic material to which the resulting prediction model is to be applied.

**Electronic supplementary material:**

The online version of this article (10.1007/s00122-019-03327-y) contains supplementary material, which is available to authorized users.

## Introduction

Genomic selection in plant breeding offers several routes for increasing the genetic gain or efficiency of plant breeding programmes (e.g. Bernardo and Yu 2007; Hickey et al. 2014; Gaynor et al. 2017). Genomic selection-based strategies can achieve this by reducing breeding cycle time, increasing selection accuracy and increasing selection intensity, three of the four factors in the breeder’s equation. Genomic prediction can reduce breeding cycle time because individuals can be selected and crossed without being phenotyped. It can increase the selection accuracy because genomic data enable more powerful statistical models and experimental designs using more observations than that can be phenotyped in a single trial round. By reducing the cost of evaluating individuals via reducing the numbers phenotyped and/or reducing their replication, application of genomic selection can increase selection intensity. A final advantage is that the prediction models may be cumulatively updated with data of trials from previous years and become more accurate, enabling individuals to be “evaluated” across a broader range of environments and years.

In livestock, there is empirical evidence of increased rates of genetic gain from the use of genomic selection to target different aspects of the breeder’s equation. For example, the first seven years of genomic selection in US dairy cattle has delivered ~ 50–100% increases in rates of genetic gain (García-Ruiz et al. 2016). Much of this gain has emanated from a reduction in generation interval. In commercial pig breeding, genomic selection has driven a 35% increase in rate of genetic gain in the breeding programme that supplies the genetics in 25% of the intensively raised pigs globally. This gain came from increased accuracy of selection and a better alignment of selection accuracy with the breeding goal (W. Herring, personal communication).

Genomic selection uses genotype data to calculate the realised relationship between individuals and in a standardised statistical framework uses data from phenotyped relatives to estimate genetic values of the selection candidates. The usefulness of genomic selection to a breeder is a function of its accuracy. This is affected by the relatedness between the phenotyped individuals in the training set and the individuals that are to be predicted (Habier et al. 2007, 2010; Meuwissen 2009; Clark et al. 2012; Hickey et al. 2014; Liu et al. 2016), which may or may not be phenotyped themselves. In addition to the level of relatedness, the sample size of the phenotyped individuals is an important factor in determining accuracy (Zhang et al. 2017).

In summary, small numbers of close relatives and very large numbers of distant relatives enable accurate predictions. Small or modest numbers of distant relatives do not enable accurate predictions, as they share only a small proportion of genome with the selection candidates and thus provide less reliable predictions (de los Campos et al. 2013). Finally, the training set should also comprise a diverse set of individuals to produce reliable predictions (Calus 2010; Pszczola et al. 2012; Pszczola and Calus 2015), as supported by recent research in both cattle (Jenko et al. 2017) and simulated barley (Neyhart et al. 2017).

The objective of this study was to explore the effect of level of relatedness between training set and validation set on genomic prediction accuracy using data from a large set of field experiments. To do this, 44 bi-parental or three-way crosses were obtained from four commercial wheat breeders in the UK, as described for the GplusE project (Mackay et al. 2015). The crosses had different degrees of relatedness among each other, and there were many shared parents. Sixty-eight F_2:4_ lines from each cross were genotyped and phenotyped for yield. As this data set is of substantial size, it enabled genomic predictions while masking specific fractions to assess the impact on genomic selection accuracy of training sets (1) of different sizes and (2) that comprise close or distant relatives, or combinations thereof.

## Materials and methods

### Germplasm

Thirty-nine bi-parental and 5 tri-parental populations were used to develop 2992 F_2:4_ lines (68 per cross). The parents of these populations were elite breeders’ germplasm consisting of both hard and soft winter wheat cultivars adapted to the UK. A total of 27 parents were used, of which 5 parents were used in 6 or more crosses, 6 parents were used in 3 or 4 crosses, and 1 parent was used in 2 crosses. The remaining 15 parents were only used in a single cross.

### Genotype data

The F_2:4_ lines were genotyped using the Wheat Breeders’ 35 K Axiom array (Allen et al. 2016). The DNA for genotyping was obtained by bulking leaves from approximately six F_4_ plants per F_2:4_ line. Genotype calling was performed using the Axiom Analysis Suite 2.0 with a modified version of the “best practices” workflow. To allow the genotype processing in the pooled genotype set-up, quality control threshold was reduced to 95 (97 normally), plate pass per cent was changed to 90 (95 normally), and average call rate was changed to 97 (98.5 normally). After quality control and genotype calling, a total of 35,143 markers were brought forward with 24,498 segregating in the 44 crosses.

### Phenotype data

The F_2:4_ lines and agronomic checks were evaluated in 2 by 4 m harvested plots at two locations (Cambridge, UK, and Duxford, UK) in the 2015–2016 growing season, and two locations (Hinxton, UK, and Duxford, UK) in the 2016–2017 growing season. All locations were managed for optimal yield by following best agronomic practice. All F_2:4_ lines were evaluated in 4 plots. Seed for eleven of the populations was unavailable in the 2015–2016 growing season. To accommodate these populations and keep the number of plots per line constant, an allocation of F_2:4_ lines was devised that was highly unbalanced across both years and locations as described below.

In the 2015–2016 growing season, 33 of the 44 populations were planted at two locations (Table 1). The experimental design for both locations was a modified α-lattice design (Patterson and Williams 1976). The design consisted of a traditional, replicated α-lattice design with un-replicated lines added to the sub-blocks. The replicated portion of the alpha-lattice design was composed of the agronomic checks and half of the lines (34) from 22 of the F_2:4_ populations. These lines were planted in two blocks split into 151 sub-blocks each containing five lines. The remaining F_2:4_ lines were randomly allocated to sub-blocks, bringing the total number of lines per sub-block to either 9 or 10. Half of the F_2:4_ lines used for the replicated portion of the design differed between locations. Thus, lines from 22 of the F_2:4_ populations were evaluated in three plots split across both locations and the lines from the remaining populations were evaluated in two plots split across locations.

**Table 1:** Trial design summary showing number of plots per tested line per location

# Lines	2015/2016		2016/2017	
	Cambridge	Duxford	Duxford	Hinxton
367	2	1	1	0
381	2	1	0	1
381	1	2	1	0
367	1	2	0	1
748	1	1	1	1
748	0	0	2	2
**Total plots**	**2992**	**2992**	**2992**	**2992**

All 44 populations were planted in the 2016–2017 growing season at two locations (Table 1); the experimental design was similar as in the previous season. The replicated portion of the α-lattice design was composed of the agronomic checks and the F_2:4_ lines from the 11 populations not planted in the 2015–2016 growing season. These lines were planted in two blocks split into 156 sub-blocks each containing five lines. Additional F_2:4_ lines from the other populations were randomly allocated to sub-blocks, bringing the total number of lines per sub-block to 10.

### Yield trial analysis

Yield phenotypes were spatially adjusted for each trial separately. An AR1 × AR1 model (Gilmour et al. 1997) was used to adjust spatial variation across both columns and rows as implemented in ASREML 3.0.22 (Gilmour et al. 2009). A summary of line means after adjusting for spatial effects is shown in Table 2.

**Table 2:** Summary of line means per location after adjusting for spatial effects

		No. of lines	Avg. value	Coef. variation (%)	Correlation^a^
2016	Cambridge	2247	8.58	6.1	0.63
2016	Duxford	2248	10.82	6.3	0.81
2017	Hinxton	2249	4.64	10.3	0.71
2017	Duxford	2235	8.24	6.6	0.62

^a^Correlation between moisture corrected yield values and spatially adjusted values

Best linear unbiased estimates (BLUEs) for each line were estimated collectively across all trials by fitting the following model:

1 $${\mathbf{y}} = {\mathbf{Xb}} + {\mathbf{u}} + {\mathbf{e}},$$

where ${\mathbf{y}}$ was the response vector of spatially adjusted yield values, ${\mathbf{b}}$ site-specific means with design matrix ${\mathbf{X}}$, ${\mathbf{u}}$ line BLUEs to estimate, and ${\mathbf{e}}$ the model residual.

### Genomic prediction

This study used the genomic best linear unbiased prediction (GBLUP) model to estimate heritabilities and predict line effects. The GBLUP model used is:

2 $${\mathbf{y}} = \mu + {\mathbf{g}} + {\mathbf{e}},$$

where ${\mathbf{y}}$ was the response vector of yield BLUEs, $\mu$ the model intercept, ${\mathbf{g}}$ the vector of genetic values of genotyped F_2:4_ and ${\mathbf{e}}$ the model residual. We assumed that ${\mathbf{g}} \sim N\left( {0, {\mathbf{G}}\sigma_{g}^{2} } \right)$ with genomic relationship matrix calculated as ${\mathbf{G}} = {\mathbf{WW^{\prime}}}/2\sum {\text{p}}_{\text{i}} \left( {1 - {\text{p}}_{\text{i}} } \right)$ (VanRaden 2008) from the centred genotype matrix ${\mathbf{W}}$ and allele frequencies ${\text{p}}_{\text{i}}$ estimated in the data set. Further, we assumed that ${\mathbf{e}} \sim N\left( {0, {\mathbf{I}}\sigma_{e}^{2} } \right)$, which was assumed uncorrelated to ${\mathbf{g}}$.

The Average-Information Restricted Maximum Likelihood (AI-REML) algorithm (Madsen et al. 1994; Johnson and Thompson 1995), as implemented in DMU v. 5.1 (Madsen and Jensen 2000), was used to fit the GBLUP model to a subset of the data (training set) and predict line effects (${\hat{\mathbf{g}}}$) in the validation set. We defined convergence of the AI-REML algorithm based on the change of variance components, $\left| {\theta^{{\left( {t + 1} \right)}} - \theta^{\left( t \right)} } \right| < 10^{ - 5}$, where $\theta^{\left( t \right)}$ is the vector of normalised variance components estimated at step $t$ (Jensen et al. 1997).

The heritability was calculated from the trial yield data per plot as $\bar{H}^{2} = \frac{{\sigma_{g}^{2} }}{{\sigma_{g}^{2} + \frac{v}{n}}}$ in which n is the number of locations in which the genotype was observed (Piepho and Mohring 2007).

### Prediction accuracies

We applied several cross-validation strategies for investigating prediction accuracies of genomic selection with varying training set sizes and grouping of training sets and validation sets, as described in detail in the following sections. In all strategies, the GBLUP model was used as described above. The prediction accuracies were calculated as the Pearson correlation (*ρ*) between the yield BLUEs and its prediction from the GBLUP model.

### Cross-validation prediction accuracy

**Fig. 1:**
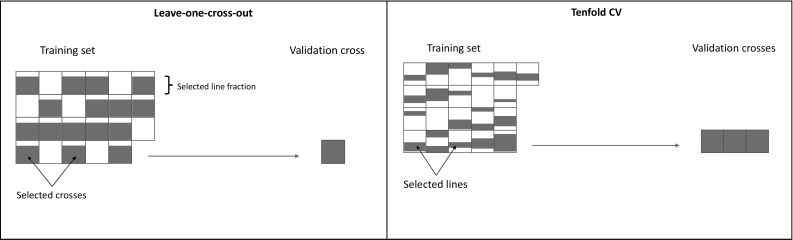
Resampling strategies applied to assess the impact of training set design. Leave-one-cross-out strategy (left) tests the impact of inclusion of the amount of crosses as well as training set size, while the tenfold cross-validation (right) tests training set size only

In the first approach, we used tenfold cross-validation and leave-one-cross-out cross-validation (effectively 44-fold cross-validation; refer to Fig. 1). Populations were randomly assigned to either training or validation set, without considering that some crosses are more closely related due to sharing a parent or other ancestors. The validation sets were entire populations, which means that line means of a population was confined entirely to either training set or validation set. Prediction accuracies were summarised on a per-cross basis. For the tenfold cross-validation, 10 replicates were performed where the tenfolds were re-sampled.

To evaluate the effect of training set size, the above two cross-validation methods were repeated using a subset of the total training set. For the tenfold cross-validation, 10%, 20%, …, 80%, 90% of records were randomly removed from the training set, before estimating variance components and predicting line means of the validation set. For each replicate and the proportion of training set masked, 10 repetitions were performed. For the leave-one-cross-out cross-validation, 1–10, 15, 20, 30, 40 crosses were randomly sampled to be used as training set. For each number of crosses sampled as training sets, 10, 20, …, 60, 65 records from each cross were sampled. Again, 10 repetitions were performed. We emphasise that the validation sets were always entire populations (from 3 to 4 crosses in tenfold cross-validations, from single cross in leave-one-cross-out), and no records of the validated populations were included in the training set.

### Prediction accuracy with related or unrelated crosses

In the second approach, we evaluated the prediction accuracies under different levels of relatedness between validation and training sets. The 6 crosses of the 4 most frequently used parents were targeted as validation crosses and tested separately. In summary, the training sets consisted of varying proportions of sister lines and half-sibs from offspring of either one or both parents or unrelated crosses. Specifically, for each validation cross, training sets were designed to consist of either one or several crosses of one parent, an equal number of crosses from each parent, nominally unrelated crosses, or equal number of related and unrelated crosses. To reduce computation time, for each training set of crosses, 5 combinations were sampled from the large number of possible combinations. For each training set, the validation cross contributed with 0, 1, 2, or 3 quarters of its lines. The prediction accuracies were evaluated for the fourth quarter of lines that were not used in the training set. For each combination of training set, 10 replicates were performed as well as cycling through all four quarters of the validation cross as training set.

## Results

Forty-four bi- and tri-parental crosses from 27 parents were analysed for yield with a GBLUP model (1), using BLUEs from 4 trials (2 trials in 2016 and 2 trials in 2017).

### Trait heritability

**Fig. 2:**
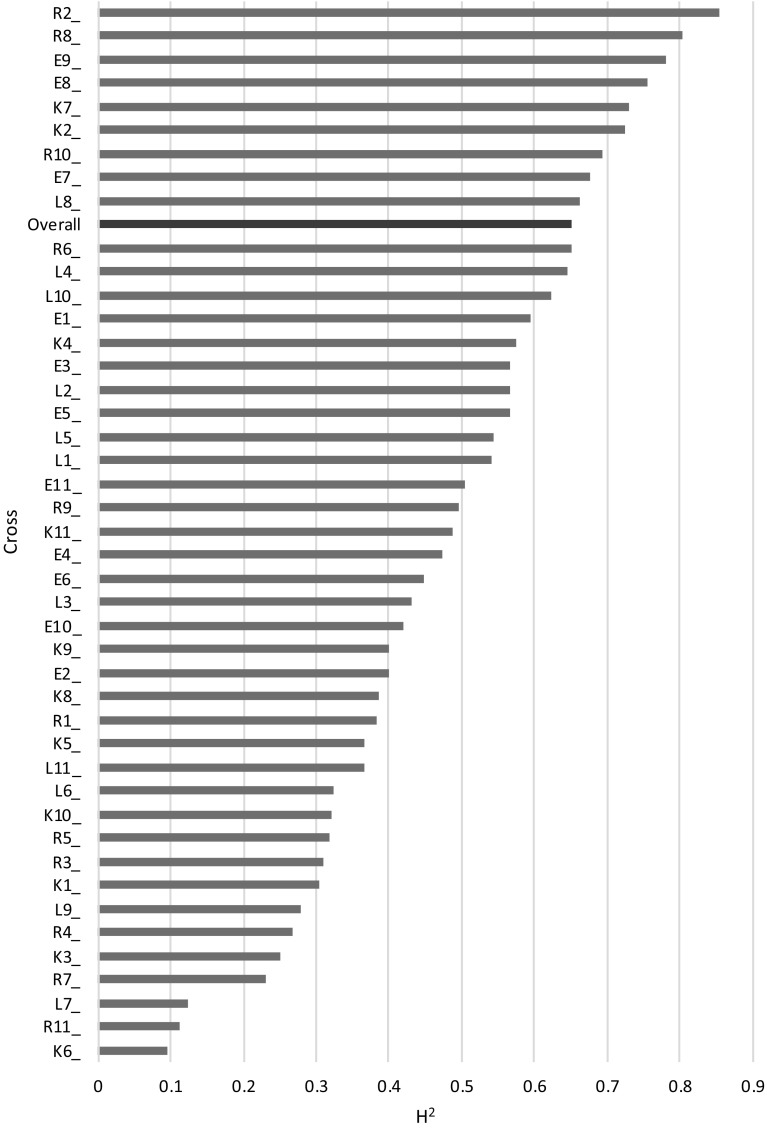
Yield heritabilities when estimated per cross. Crosses (blue bars) are ordered by heritability value; overall heritability for this trait is shown in red

The overall heritability of yield for all populations over all four trial locations was estimated at 0.65. Heritabilities estimated on a single cross were highly variable, ranging from as low as 0.1 to as high as 0.85 (Fig. 2).

### Cross-validation prediction accuracy

Prediction accuracies were 0.125–0.127 using two different cross-validation approaches (Table 3). In these two approaches, all lines of the crosses used for validation were absent from the training set. Using a tenfold cross-validation approach where individual lines, not all lines of a cross, were selected for validation sets, the prediction accuracy was slightly higher (0.142) when calculated on a per-cross basis (“tenfold, random”, Table 3). The prediction accuracy was higher when calculated across all crosses in the validation set, due to capturing variation within and between crosses (0.289 and 0.543, Table 3). In general, the prediction accuracies of tri-parental crosses are higher than those of bi-parental crosses, although there is large variation within each of these two family groups (Table 3, last column).

**Table 3:** Prediction accuracies using the largest training sets by cross-validation approach

	Correlation metric	Training set size	Correlation^a^	Bi-/tri-parental^d^
Leave-one-cross-out	By cross	2787	0.127 _0.222_	0.12 _0,.20_/0.20 _0.11_
Tenfold, crosses	By cross	2563	0.125 _0.193_	0.11 _0.20_/0.20 _0.08_
Tenfold, random^b^	By cross	2567	0.142 _0.195_	0.12 _0.17_/0.24 _0.09_
Tenfold, crosses	Across all^c^	2567	0.289 _0.259_	N/A
Tenfold, random^b^	Across all^c^	2567	0.543 _0.009_	N/A

^a^Average across all replicates. Small font displays inter-quantile range for correlations

^b^Tenfold cross-validation where validation and training sets were grouped by lines instead of crosses

^c^Correlations were calculated across multiple crosses in validation set

^d^Average correlation (^a^), but across bi-parental or tri-parental crosses

**Fig. 3:**
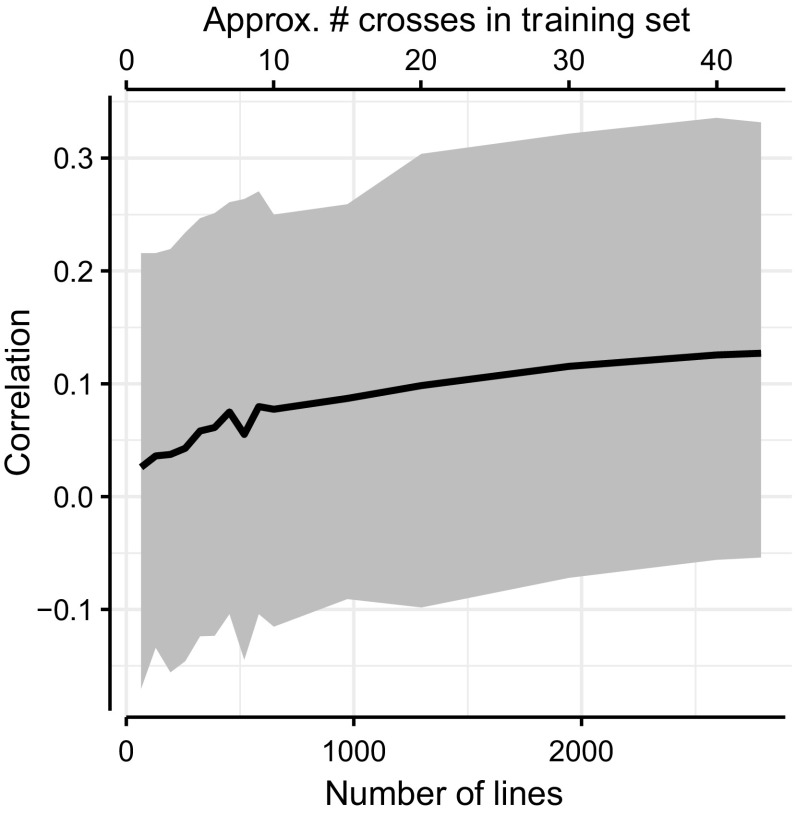
Increasing training set size increased prediction accuracy (correlation). Solid line shows average of all leave-one-cross-out cross-validations with 10th and 90th percentile range shown by greyed area

The prediction accuracy was found to increase with training set size. Figure 3 displays the average prediction accuracy across all crosses with 10th and 90th percentile range shown as the greyed area. The prediction accuracy varied greatly between the crosses (Supplemental Fig. 1) with some accuracies as high as 0.45 (cross 7) and as low as − 0.20 (cross 30). For 31 crosses out of 44, significant positive prediction accuracies were found (Wald’s test, *p* < 0.05). Crosses with higher phenotypic variance generally yielded higher predictions; in Supplemental Figure 1, prediction accuracy plots for individual crosses are sorted with decreasing phenotypic variance. Finally, the two cross-validation approaches generally produced similar results (Supplemental Figure 1), but when the training sets were small, the accuracy of predictions from leave-one-cross-out was less stable than from tenfold cross-validation. The leave-one-cross-out sampled entire crosses in contrast to the tenfold cross-validation, where lines across all crosses except the validated cross were sampled.

**Fig. 4:**
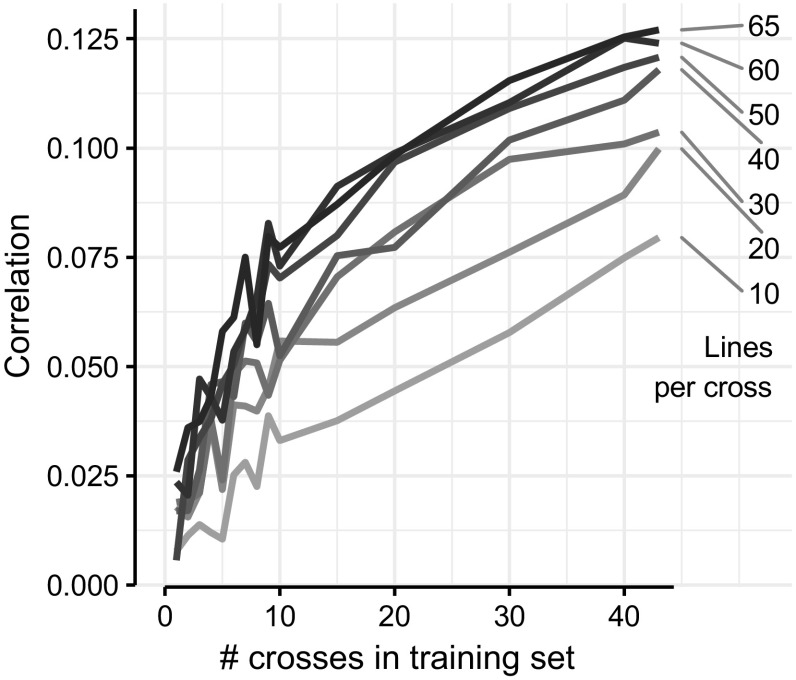
Prediction accuracies increased with increasing number of crosses or increasing number of lines per cross in training set. Right-hand numbers show number of lines per cross in training set

The prediction accuracy increased with an increasing number of crosses in training set or increasing number of lines per cross in training set. Figure 4 displays the average prediction accuracy when sampling a number of lines from a number of crosses (*x*-axis). Adding an additional 10 or 15 lines to a training set of 50 lines per cross generally led to a low increase in prediction accuracy as compared to adding them to training sets of ≤ 40 lines per cross, irrespective of the number of crosses included in the training set. Increments of accuracy by adding more lines, tested with a *t* test, were found to be not significant (*p* < 0.05).

All accuracies are based on the prediction of random members of each family, independent on their relative position in the performance distribution within the cross. It was observed that for the prediction of the highest performing 15 lines in each cross, which are expected to include the most relevant genotypes for a breeder, the prediction accuracy was close to zero (not shown).

### Prediction accuracies with related or unrelated crosses

**Fig. 5:**
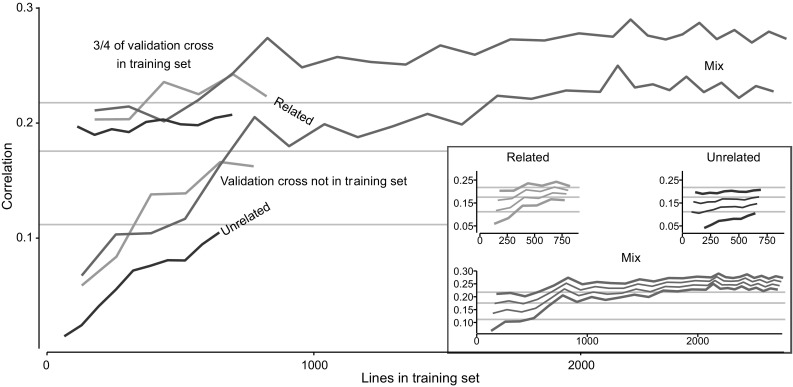
Prediction accuracies increased when the validation cross was partly in training set or had its related crosses in training set. Results show average prediction accuracies for six validation crosses. Lines show prediction accuracies when training set is comprised of related crosses (green solid line), unrelated crosses (purple line), or a mix of both (blue line). Lower set of lines show prediction accuracies when validation crosses were not included on the training set; upper set of lines show prediction accuracies when validation crosses were included in the training set with 3/4 of lines. Grey horizontal lines show average prediction accuracy using *only* 1/4, 2/4 or 3/4 of validation cross as training set. Inserted figure shows the increase in accuracy when adding 1/4, 2/4 and 3/4 of the validation group to the training set. The thick lines in the inserted figure denote the lines of the main figure (color figure online)

Using related crosses as a training set generally resulted in higher prediction accuracies than using unrelated crosses. This is shown in Fig. 5, where the green lines (related training sets) are above the purple lines (unrelated training sets). Using both related and unrelated crosses in equal proportions (blue lines, Fig. 5) led generally to similar correlations to those for related crosses. At approximately 700 to 800 lines in the training set, the prediction accuracy using both related and unrelated crosses plateaued; this was where additional crosses in the training set were unrelated to the validation cross. The level of prediction accuracy of the training set comprising both related and unrelated crosses (lower blue line, Fig. 5) was higher than that in Fig. 3 because results in Fig. 5 are averages over just 6 crosses rather than over all crosses as in Fig. 3.

Using only 1, 2, or 3 quarters of the validation cross as training set (grey, horizontal lines, Fig. 5) generally led to prediction accuracies that were higher than using a few unrelated or related crosses as the training set. Adding three quarters of the validation cross to the training sets of other crosses generally increased the prediction accuracy, as shown with the upper thick lines in Fig. 5. The gradual increase in prediction accuracy when adding 1, 2, or 3 quarters of the validation cross to the training set is shown in the inserted plot in Fig. 5.

## Discussion

In this study, we have demonstrated the impact of training set size and relatedness on genomic prediction in wheat, using F_2:4_ lines from 44 bi- and tri-parental crosses. The results were consistent with expectations from existing literature (as discussed in the next sections). Specifically, we found that increasing the size of the genomic prediction training set increased accuracy. We also found that training sets composed of lines more closely related to the validation set produce higher prediction accuracies than equivalently sized training sets of more distantly related lines.

It is important for genomic prediction of a complex trait that it displays a reasonable heritability. Our estimate of broad sense heritability for yield (0.65) is well within the range of similar studies in wheat (Poland et al. 2012; Combs and Bernardo 2013; Michel et al. 2016; Schopp et al. 2017; Norman et al. 2017). We note that the heritability values within individual families (Fig. 2) cover the whole range of heritability for this trait reported in the literature.

The various strategies of data subset masking applied in this study have enabled us to demonstrate both training set size and relatedness as parameters that influence successful genomic prediction. Generally, increasing the training set size increased the prediction accuracy, as expected from existing theory (Daetwyler et al. 2008; Goddard 2009; Hickey et al. 2014) and field reports (Liu et al. 2016; Zhang et al. 2017). However, we can add three observations that put some nuance to this general conclusion. First (1), with a fixed training set size, it is better to increase the number of populations (crosses) rather than number of lines per population (cross). Second (2), the prediction accuracy plateaus when adding additional crosses that are unrelated to the predicted cross (Figure). Third (3), prediction accuracies vary greatly between individual crosses, and this could not be explained by either the crosses’ phenotypic variance or heritability.

For item (1), we showed that, for example, using 10 crosses with 40 lines per cross gave prediction accuracy of ≈ 0.06, while 40 crosses with 10 lines per cross gave prediction accuracy of ≈ 0.075 (Fig. 3). We assume that in both strategies different processes increase the accuracy with the addition of extra lines: In the first case, entire crosses were masked simulating the future prediction of an unphenotyped cross. In comparison, increasing the number of lines instead of number of crosses (while constraining the training set size) did not necessarily improve the prediction accuracy. The lines capture the crosses’ variance, and there will be a limit to how much more variance additional lines will capture, hence no additional gain. The exception to this was adding fractions of the validation cross’ lines to the training set (Fig. 5).

For item (2), we saw in figure that using training sets comprised of exclusively unrelated crosses resulted in lower prediction accuracies than training sets that included related crosses. Using training sets comprised of either exclusively related crosses or related and unrelated crosses (half-and-half) both resulted in approximately the same prediction accuracy. The comparison between these three sets stops at about 800 lines in the training set, because beyond this point, additional crosses were no longer distinctively related or unrelated. Therefore, after this point the slope of increase in prediction accuracy is less steep, as the crosses added to the training set are less related.

For item (3), there was no observable connection between how well the cross could be predicted and the cross’ heritability or the observed phenotypic variance. Likewise, these values did not correspond to how well the data from the cross could be used to predict breeding values in other crosses.

It should be noted that all observed absolute prediction accuracies in this study are rather low, which is probably mainly caused by the pooled strategy used for the genotyping. In a separate study, it was demonstrated that higher prediction accuracies (with equivalent correlation values in the range of 0.6–0.9) could be found using other type of predictor data, collected with large-scale phenotypic trait collection technology (Buntjer et al. in preparation). The overall low correlation values found with the genomic predictions in this study suggest generalisation of the observed trends should be taken with care.

One of the major practical implications of this study is that increased prediction accuracies can be obtained by balancing the training set for genomic selection with phenotypic and genomic data of multiple related crosses, which could be taken into account in advance when designing the training population (as earlier proposed by Rincent et al. 2012). For existing data sets, a strategy may be applied of supplementing these with phenotypic data from previous trials (provided genotype-by-environment interaction is limited or can be accounted for by use of trait data for control lines). Although such data might be present within the context of a rolling breeding programme, obtaining genomic data presents a bottleneck as this requires genotyping of (old) biological material that might not be readily available and will require investment in at least low-density genotyping. In case high-density genotype data sets are available for the parental lines, high-density genotype information for their offspring populations can subsequently be obtained by imputation, as reported by Hickey et al. (2015) and Gorjanc et al. (2017).

## Conclusions

Genomic predictions of yield across 44 populations resulted in modest correlations between observed and predicted values. The correlations did increase with training set size, but by selecting training sets that comprised related crosses improved the correlation more than increasing training set size. The results also showed that if the training set size is fixed, using few lines from more crosses, rather than many lines from few crosses, resulted in higher correlations.

### Author contribution statement

Wheat crosses were made by JL, EB, CB, PJ, SB, EF, BP, SS and CH; wheat yield trials were conducted by RJ, PH, EO and IJM; ARB co-ordinated genotyping; SME, RCG and GG performed data analysis; SME, JBB, RCG and JMH wrote the manuscript; JMH and IJM conceived the study, designed the experiment and led the project.

## Electronic supplementary material

Below is the link to the electronic supplementary material.

Supplemental Figure 1: Per-cross correlation under two approaches (A), ordered by decreasing variance of crosses’ BLUEs (B). Grey, horizontal lines are guides for zero correlation (dashed) and overall average correlation of 0.127 (solid). Crosses in (A) are ordered with decreasing variance of their BLUEs, same order as in (B). (DOCX 56 kb)
