# Supplementary material for: The effects of training population design on genomic prediction accuracy in wheat
Source: Theor Appl Genet. 2019 Mar 19;132(7):1943–52. doi: 10.1007/s00122-019-03327-y (PMC6588656; doi:10.1007/s00122-019-03327-y)
Supplement: Supplementary file 1 — Supplemental Figure 1: Per-cross correlation under two approaches (A), ordered by decreasing variance of crosses’ BLUEs (B). Grey, horizontal lines are guides for zero correlation (dashed) and overall average correlation of 0.127 (solid). Crosses in (A) are ordered with decreasing variance of their BLUEs, same order as in (B). (DOCX 56 kb) [file 122_2019_3327_MOESM1_ESM.docx]

Supplemental figure 1: Per-cross correlation under two approaches (A), ordered by decreasing variance of crosses’ BLUEs (B). Grey, horizontal lines are guides for zero correlation (dashed) and overall average correlation of 0.127 (solid). Crosses in A) are ordered with decreasing variance of their BLUEs, same order as in B).
